# Supplementary material for: Flow interactions lead to self-organized flight formations disrupted by self-amplifying waves
Source: Nat Commun. 2024 Apr 24;15:3462. doi: 10.1038/s41467-024-47525-9 (PMC11043384; doi:10.1038/s41467-024-47525-9)
Supplement: Supplementary file 2 — Description of Supplementary Information [file 41467_2024_47525_MOESM2_ESM.pdf]

**Description of Supplementary Files for**
**“Flow interactions lead to self-organized flight formations disrupted by self-amplifying waves”**

File Name: **SourceData**
Description: This spreadsheet file contains pages that provide all the source data plotted in the figure panels.

File Name: **Movie 1**
Description: A live recording of the experimental apparatus in operation. Five foils are driven to flap up and down in unison, and they freely and interactively propel around a water tank.

File Name: **Movie 2**
Description: Top-view recording of a self-organized flock in experiments. The foils assemble into a lattice with regular gap spacing.

File Name: **Movie 3**
Description: Top-view recording of a collision. Strong fluctuations cause the last member to collide with its upstream neighbor, and the flock subsequently collapses.

File Name: **Movie 4**
Description: Reanimation of experimental measurements of the dynamics of five foils. Their rotational propulsion around a tank is displayed as translation.

File Name: **Movie 5**
Description: Animation of simulations corresponding to five foils traversing a periodic domain. The wake signal is represented by arrows trailing each member.

File Name: **Movie 6**
Description: Animation of simulations of five foils in an open domain and with the leader given an oscillatory perturbation.

File Name: **Movie 7**
Description: Animation of simulations of 30 foils. The leader is given oscillatory perturbations throughout the run, and the fluctuations amplify as they travel down the group. The simulation terminates with a collision among later members.

File Name: **Movie 8**
Description: Experimental recording of a flock with a vacancy defect. A void between members 3 and 4 is stably maintained.

File Name: **Movie 9**
Description: Animation of simulations of 10 foils flapping in phase and with the leader given an oscillatory perturbation.

File Name: **Movie 10**
Description: Animation of simulations of 10 foils flapping in anti-phase and with the leader given an oscillatory perturbation.
