# Supplementary figures and images for: Flow interactions lead to self-organized flight formations disrupted by self-amplifying waves
Source: Nat Commun. 2024 Apr 24;15:3462. doi: 10.1038/s41467-024-47525-9 (PMC11043384; doi:10.1038/s41467-024-47525-9)

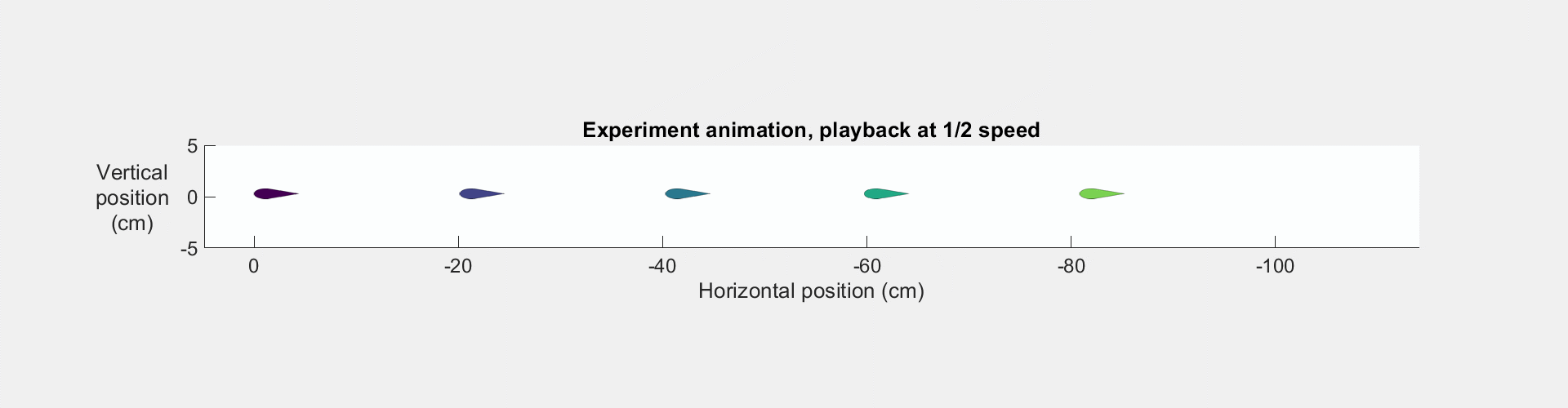

Supplement: Supplementary file 6 — Movie 4 [file 41467_2024_47525_MOESM6_ESM.gif]

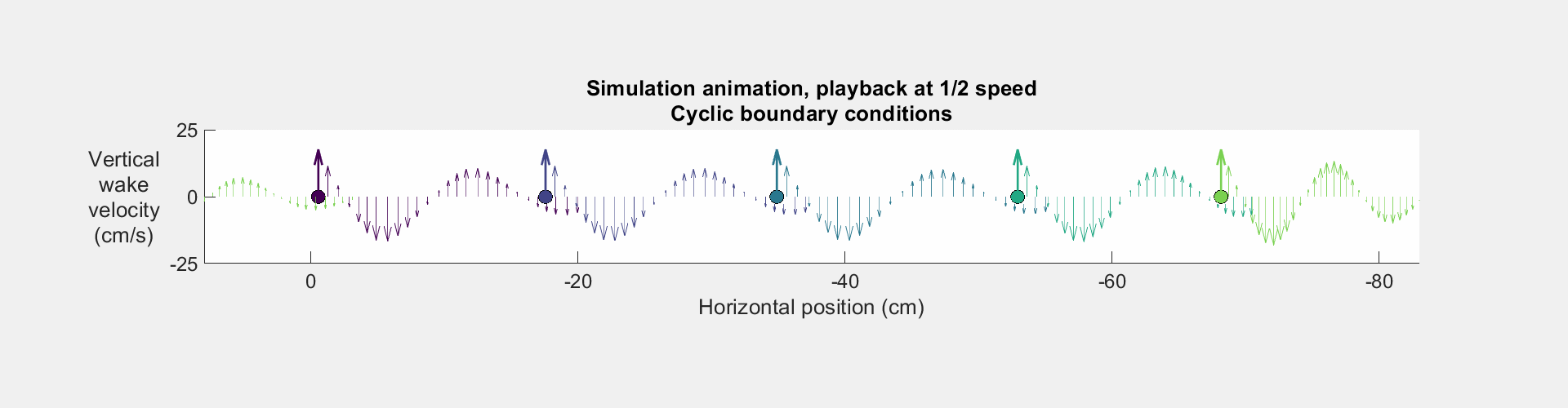

Supplement: Supplementary file 7 — Movie 5 [file 41467_2024_47525_MOESM7_ESM.gif]

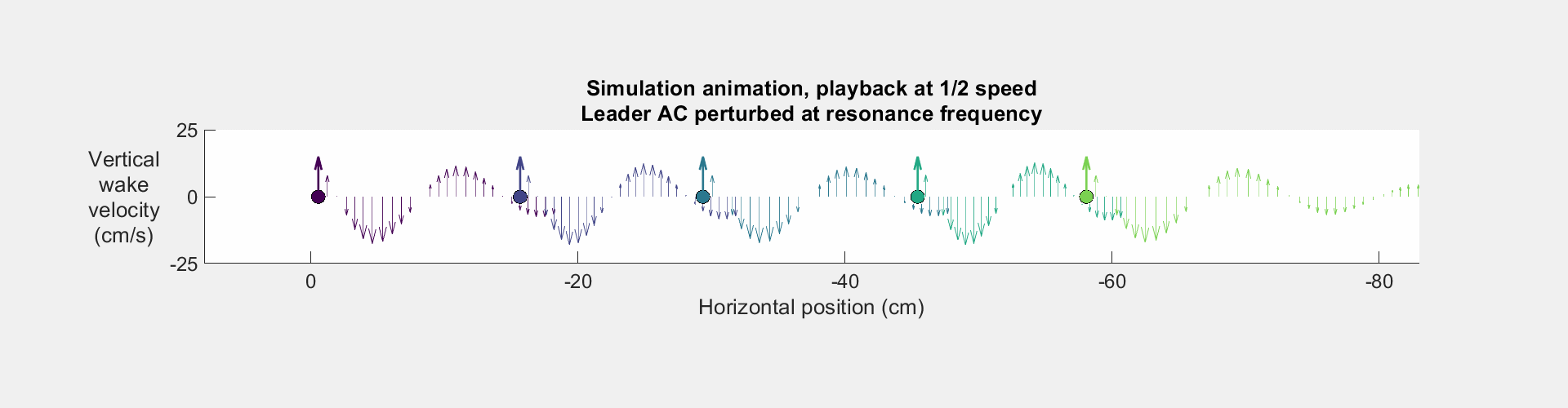

Supplement: Supplementary file 8 — Movie 6 [file 41467_2024_47525_MOESM8_ESM.gif]

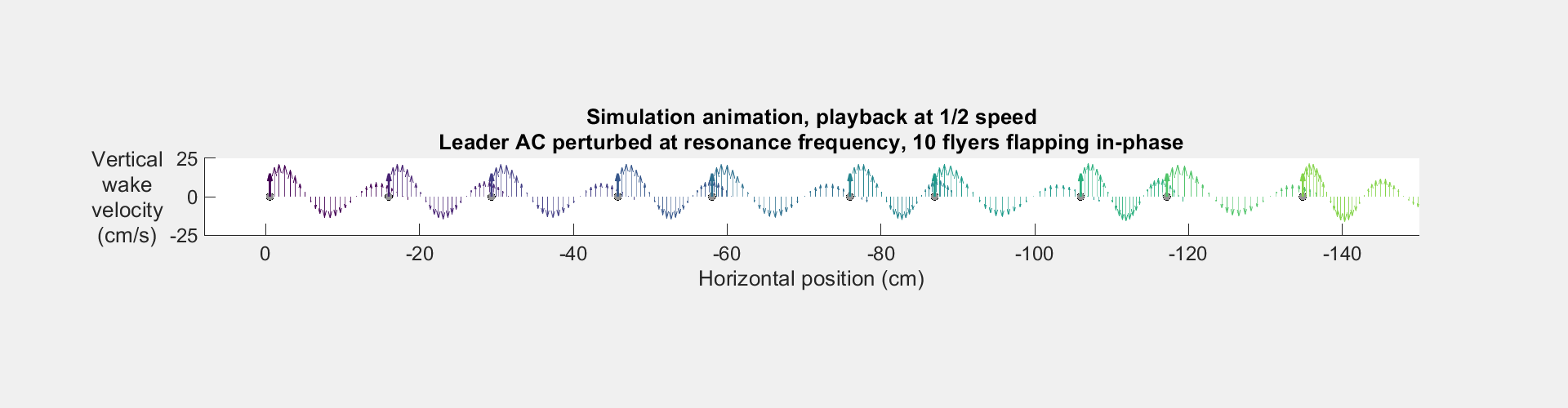

Supplement: Supplementary file 11 — Movie 9 [file 41467_2024_47525_MOESM11_ESM.gif]

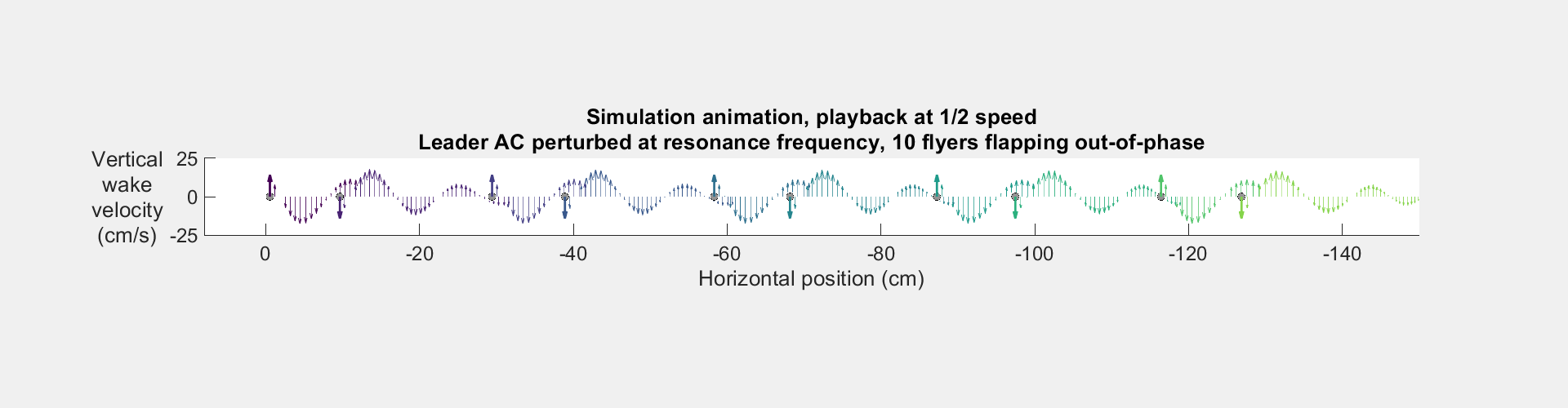

Supplement: Supplementary file 12 — Movie 10 [file 41467_2024_47525_MOESM12_ESM.gif]
